# 

**Published:** 1915-12

**Authors:** 


### CONTENTS.

ORIGINAL COMMUNICATIONS—

Traumatic Visceral Pneumothorax Resulting From Artificial Pneumothorax, by Stevens T. Harris, M. D., Atlanta, Ga. ........ 415

A Clinical Lecture on Gastric and Dudodenal Ulcer, by J. L. Campbell, M. D., F. A. C. S. ........ 420

Syphilitic Serpiginous Ulceration of the Rectum and Buttocks, by E. M. Merritt, M. D., Atlanta, Ga.. 432

EDITORIAL ........ 434

SELECTIONS AND ABSTRACTS ........ 437

BOOK REVIEW ........ 461

